# Functional Characterisation of Surfactant Protein A as a Novel Prophylactic Means against Oncogenic HPV Infections

**DOI:** 10.3390/ijms25147712

**Published:** 2024-07-14

**Authors:** Sinead Carse, Tim Reid, Jens Madsen, Howard Clark, Artur Kirjakulov, Martina Bergant Marušič, Georgia Schäfer

**Affiliations:** 1 International Centre for Genetic Engineering and Biotechnology (ICGEB), Observatory, Cape Town 7925, South Africa; carsesinead@gmail.com; 2 Institute of Infectious Disease and Molecular Medicine (IDM), Faculty of Health Sciences, University of Cape Town, Observatory, Cape Town 7925, South Africa; tim.reid@uct.ac.za; 3 Division of Medical Biochemistry, Department of Integrative Biomedical Sciences, University of Cape Town, Observatory, Cape Town 7925, South Africa; 4 South African Tuberculosis Vaccine Initiative (SATVI), University of Cape Town, Observatory, Cape Town 7925, South Africa; 5 Targeted Lung Immunotherapy, Neonatology, Institute for Women’s Health, University College London, London WC1E 6BT, UK; jens.madsen@ucl.ac.uk (J.M.); h.clark@ucl.ac.uk (H.C.); 6 Infection, Inflammation and Repair, Faculty of Medicine, University of Southampton, Southampton General Hospital, Southampton SO16 6YD, UK; kirjakulov@gmail.com; 7 Laboratory for Environmental and Life Sciences, University of Nova Gorica, Vipavska 13, 5000 Nova Gorica, Slovenia; martina.bergant@ung.si; 8 Wellcome Centre for Infectious Diseases Research in Africa, University of Cape Town, Observatory, Cape Town 7925, South Africa

**Keywords:** surfactant protein A (SP-A), human papillomavirus (HPV), pseudovirus, cervical cancer, HPV prophylactic, THP-1-derived immune cells, RAW264.7 murine macrophages, HaCaT keratinocytes, low- and middle-income countries (LMIC)

## Abstract

Human papillomavirus (HPV) infection poses a significant health challenge, particularly in low- and middle-income countries (LMIC), where limited healthcare access and awareness hinder vaccine accessibility. To identify alternative HPV targeting interventions, we previously reported on surfactant protein A (SP-A) as a novel molecule capable of recognising HPV16 pseudovirions (HPV16-PsVs) and reducing infection in a murine cervicovaginal HPV challenge model. Building on these findings, our current study aimed to assess SP-A’s suitability as a broad-spectrum HPV-targeting molecule and its impact on innate immune responses. We demonstrate SP-A’s ability to agglutinate and opsonise multiple oncogenic HPV-PsVs types, enhancing their uptake and clearance by RAW264.7 murine macrophages and THP-1 human-derived immune cells. The SP-A opsonisation of HPV not only led to increased lysosomal accumulation in macrophages and HaCaT keratinocytes but also resulted in a decreased infection of HaCaT cells, which was further decreased when co-cultured with innate immune cells. An analysis of human innate immune cell cytokine profiles revealed a significant inflammatory response upon SP-A exposure, potentially contributing to the overall inhibition of HPV infection. These results highlight the multi-layered impact of SP-A on HPV, innate immune cells and keratinocytes and lay the basis for the development of alternative prophylactic interventions against diverse HPV types.

## 1. Introduction

Human papillomaviruses (HPVs) are small, non-enveloped DNA viruses and represent the most prevalent sexually transmitted infection worldwide [1,2]. They pose a significant public health concern due to their robust association with various cancers, particularly cervical cancer, with HPV types 16 and 18 identified as the predominant oncogenic strains worldwide [3,4]. Cervical cancer is notably more prevalent in low- and middle-income countries (LMICs), where it is the primary cause of female cancer-related deaths, especially in Sub-Saharan Africa [5]. This is exacerbated by the concurrent Human Immunodeficiency Virus (HIV)/Acquired Immunodeficiency Syndrome (AIDS) epidemic in the region [6,7,8]. Despite the availability of highly effective prophylactic vaccines against the most prevalent high-risk HPV types, their distribution in LMICs is significantly hampered by the cost, logistical challenges in reaching the target population, cultural barriers and the need for a cold chain [9,10,11,12,13,14,15]. Consequently, there is a pressing need for alternative strategies for protecting against sexually transmitted HPVs [16].

HPV has evolved complex mechanisms for evading the host immune system, enabling it to persist, thereby increasing the risk of cancer development [17,18]. There is no viraemia upon infection, very low levels of viral proteins are expressed initially and no cell lysis occurs, resulting in little to no release of pro-inflammatory cytokines from infected keratinocytes [19]. Moreover, new viral particles are only released when infected keratinocytes have migrated to the outer epithelial layers, where immune surveillance is minimal [20]. Additionally, the HPV oncoproteins E6 and E7 inhibit key immune pathways, including the IFN response, and prevent MHC class I molecule expression, which impairs antigen presentation to cytotoxic T lymphocytes [17,21,22]. These evasion strategies challenge the induction of a robust and targeted immune response against HPV.

Innate immunity is crucial in the body’s initial defense against infections, including those caused by oncogenic HPV [23,24,25]. It involves immune cells like macrophages, dendritic cells and natural killer (NK) cells, along with pattern recognition receptors (PRRs) that detect pathogen-associated molecular patterns (PAMPs) and damage-associated molecular patterns (DAMPs) [26,27,28,29,30]. These components enable the rapid identification of viruses and altered self-cells, triggering cytokine and chemokine production to orchestrate an inflammatory response [31]. Enhancing the innate immune response to HPV could be key to preventing infection at the early stages.

We have previously identified surfactant protein A (SP-A), a key member of the collectins family within the innate immune system, as a promising prophylactic molecule for enhancing the immune response to HPV16 pseudovirus (HPV16-PsVs) infection [32]. SP-A is a large hydrophilic protein crucial for lung surfactant homeostasis and pulmonary immunity [33] but has also been found to exert its functions at non-pulmonary sites [34]. As a soluble, collagenous C-type lectin PRR that is calcium-dependent, SP-A’s ability to recognise and bind specific ligands typically occurs through its carbohydrate recognition domains (CRDs) [33]. This interaction triggers a variety of immune responses, including the agglutination of pathogens, enhanced opsonisation and phagocytosis, the regulation of macrophage function and inflammation and even direct pathogen killing [35]. Our research demonstrated that SP-A can bind to HPV16-PsVs, improving their recognition by murine immune cells in both in vitro and in vivo mouse models [32]. However, it remains to be determined whether SP-A can similarly enhance their recognition by human innate immune cells and if this leads to functional consequences regarding the infection of human keratinocytes. Furthermore, the potential of SP-A to opsonise multiple HPV types and affect their infectability has not yet been explored, highlighting the need for broader-spectrum studies. We therefore characterised SP-A’s ability to enhance the immune recognition of multiple HPV types using the human keratinocyte cell line HaCaT in the presence of SP-A and a human innate immune cell panel. We further characterised the potential of SP-A to agglutinate HPV and studied its functional consequence for cellular uptake and lysosomal accumulation. Furthermore, the modulation of cytokine expression in monocytes and dendritic cells was assessed in the presence of HPV16-PsVs and SP-A.

We identified several mechanisms that govern the functional impact of SP-A on dampening HPV infection, such as the enhanced agglutination of the virions, increased immune cell recognition and viral clearance by enhanced lysosomal degradation and the mounting of a pro-inflammatory response. These results may lay the basis for developing novel interventions that increase the innate immune response against incoming HPV, resulting in overall protection from new infections.

## 2. Results

Based on previous work demonstrating the modulatory effects of SP-A on HPV16-PsVs’ infection of murine cells, we extended the functional characterisation of the immune mechanisms associated with SP-A’s ability to recognise and bind HPV to human immune cells and keratinocytes and multiple oncogenic HPV types.

### 2.1. SP-A Agglutinates HPV Particles

**Figure 1:**
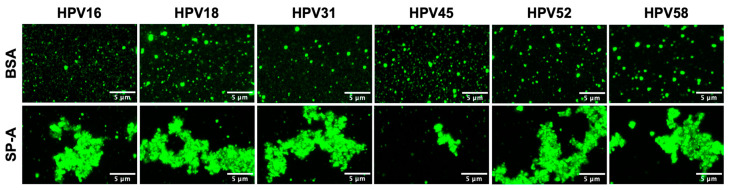
HPV-PsVs are agglutinated by SP-A to different extents. AF488-labelled HPV-PsVs were preincubated with SP-A or the BSA control for 1 h in the presence of 5 mM CaCl_2_ before imaging with a Zeiss LSM 980 confocal microscope (Carl Zeiss, Oberkochen, Germany). Shown are representative images taken at the 63x/1.4 NA oil immersion objective. Scale bar = 5 µm.

To investigate whether SP-A can aggregate HPV-PsVs, fluorescent images of SP-A-opsonised HPV-PsVs (SP-A:HPV-PsVs) complexes were captured. After incubating with SP-A and CaCl_2_ for 1 h, various oncogenic HPV-PsVs types were observed to cluster together more prominently than in the control samples treated with BSA (Figure 1). The size of clusters and thus the extent to which SP-A agglutinated HPV varied among the types, where HPV16, 18, 31 and 52 particularly formed large complexes compared to the respective BSA controls.

### 2.2. SP-A Enhances HPV16-PsVs Uptake into Immune Cells but Dampens Uptake into Epithelial Cells

**Figure 2:**
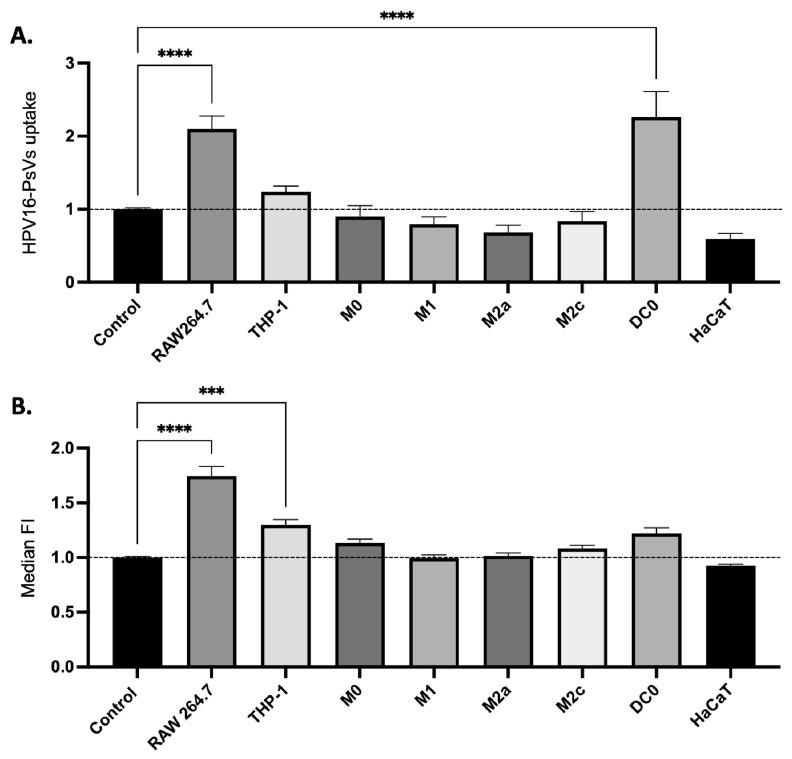
**HPV16-PsVs’ internalisation into HaCaT and various immune cells is altered by preincubation with SP-A.** Cells were incubated with HPV16-PsVs for 1 h, as described, before acquisition with a BD LSRFortessa. (**A**) Fold-change of HPV16-PsVs uptake in the presence of SP-A is depicted with the BSA control set as 1 (indicated by the dotted line). (**B**) Fold-change of the MFI of internalised HPV16-PsVs in the presence of SP-A is depicted with the BSA control set as 1 (indicated by the dotted line). Data of three independent experiments are presented relative to the uptake of the BSA control group. Statistical significance was determined by one-way ANOVA and Dunnet’s multiple comparison tests. *** = *p* < 0.001; **** = *p* < 0.0001; no symbol denotes “not significant”.

We have previously shown that HPV16-PsVs’ preincubation with SP-A results in their increased uptake into RAW264.7 murine macrophages and innate immune cells derived from naïve mouse female genital tract tissue, dampening overall infection [32]. The same approach was used to translate these findings to human innate immune cells—specifically, the populations of the innate human immune cell panel (ICP) derived from THP-1 cells and the human keratinocyte target cell line HaCaT. Confirming our previous study [32], the preincubation of HPV16-PsVs with SP-A increased the viral particle uptake in RAW264.7 murine macrophages compared to that in the BSA control, as shown by a shift in the positive fluorescent signal (Figure 2A): the number of HPV16-PsVs-positive cells increased by about 2-fold in RAW264.7 for the SP-A condition, along with a 1.75-fold increase in the median fluorescent intensity (MFI) (Figure 2B). Among the human immune cell types analysed, THP-1-derived immature dendritic cells (DC0) showed a significant increase in uptake (2-fold), while THP-1 monocytes showed a significant increase in MFI (1.3-fold). Particle uptake and/or MFI in M0, M1, M2a and M2c macrophages were not enhanced by SP-A preincubation.

Notably, the response of the keratinocyte cell line, HaCaT, to HPV16-PsVs infection yielded intriguing differences in particle uptake dynamics compared to the ICP, as a twofold decrease in HPV16-PsVs uptake in the presence of SP-A compared to that of the BSA control, with a slight decrease in the MFI, was observed. Similar results were obtained with two other epithelial cell lines, HeLa and NIKS (Figure S1). This increased uptake in immune cells and dampened uptake in epithelial cells were shown to require prior opsonisation and agglutination of the viral particles by SP-A (Figure S2).

**Figure 3:**
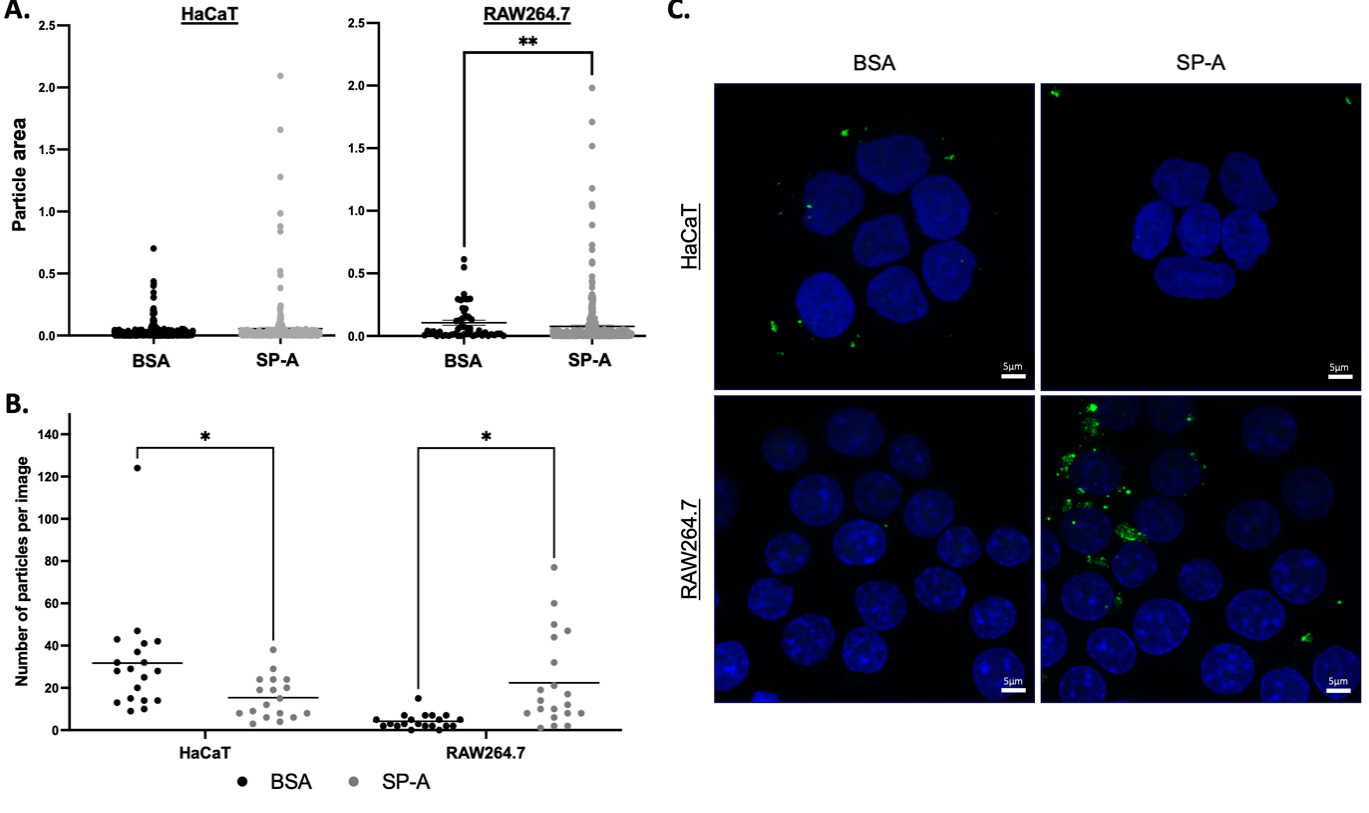
**HPV16-PsVs particle size and occurrence in HaCaT and RAW264.7 cells at 3 h post-infection.** In total, 20 z-stacks of HaCaT and RAW264.7 cells infected with BSA- or SP-A-coated HPV16-PsVs were acquired with a Zeiss LSM 980 confocal microscope. Using the Fiji “Analyze particles” function to analyse the maximum intensity projections of the z-stacks, the (**A**) particle area (in µm^2^) and (**B**) number of particles were determined. Each point on the graphs is the average particle area in pixels or the average number of particles for each image. The number of particles was normalised to cell number and cell area. (**C**) Representative confocal images of HaCaT and RAW264.7 cells infected with BSA- or SP-A-coated HPV16-PsVs taken at 3 h post-infection. Nuclei were stained with Hoechst 33342 (blue), and AF488-HPV16-PsVs are shown in green. Statistical significance was determined by one-way ANOVA and Sidak’s multiple comparison tests. * = *p* < 0.05; ** = *p* < 0.01; no symbol denotes “not significant”. Scale bar = 5 µm.

As illustrated in Figure 1, SP-A demonstrates the ability to agglutinate HPV-PsVs when preincubated in the presence of CaCl_2_. This, along with the notable enhancement in fluorescence intensity observed in selected immune cells infected with SP-A-coated HPV16-PsVs in Figure 2, prompted further investigation into these findings. Confocal microscopy was employed to compare AF488-HPV16 clusters within and surrounding cells at 3 h post-infection. As seen in Figure 3A, the mean size of the detected particles in RAW264.7 cells was greater in the BSA group, while no significant difference in the mean particle size was observed for the HaCaT cells. However, for both cell lines, the range of particle sizes in the SP-A groups was greater, and larger clusters were observed, supporting the agglutination data shown in Figure 1. Moreover, HaCaT cells showed a decrease in particle uptake in the presence of SP-A, while RAW264.7 cells displayed an increase in particle uptake at 3 h post-infection (Figure 3B), again corroborating the flow cytometry data presented in Figure 2. Representative images are shown in Figure 3C.

### 2.3. SP-A Modulates Viral Uptake in HaCaT and RAW264.7 Cells Independently of HPV Type but Selectively Affects the Uptake of Specific HPV Types by THP-1 and Dendritic Cells

**Figure 4:**
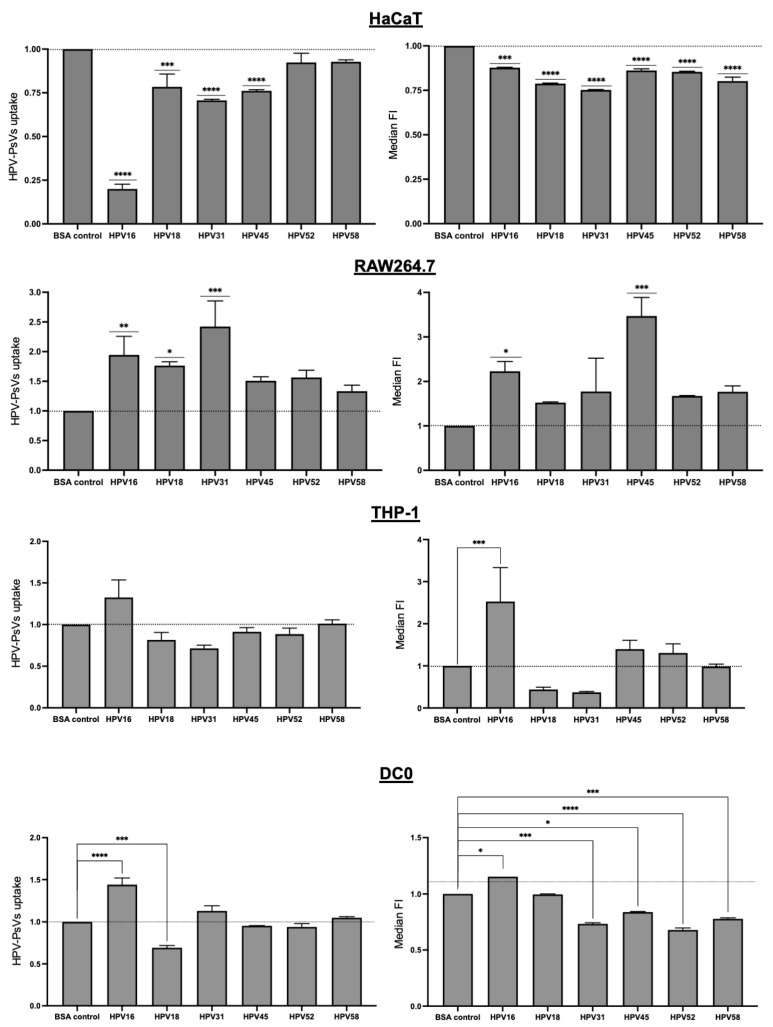
**SP-A modulates the internalisation of multiple HPV-PsVs types into HaCaT, RAW264.7, THP-1 and DC0 cells to varying degrees**. Cells were incubated with the indicated HPV-PsVs for 1 h, as described, before acquisition with a BD LSRFortessa. HPV-PsVs uptake is depicted on the left and the MFI of internalised HPV-PsVs is depicted on the right. Data of three independent experiments are presented relative to the uptake of the BSA control group, which was set as 1 (indicated by the dotted line). Statistical significance was determined by one-way ANOVA and Sidak’s multiple comparison tests. * = *p* < 0.05; ** = *p* < 0.01; *** = *p* < 0.001; **** = *p* < 0.0001; no symbol denotes “not significant”.

In order to assess the broad-spectrum capabilities of SP-A, we tested the uptake of a selection of oncogenic HPV types in HaCaT as well as RAW264.7, THP-1 and DC0 cells, as these were the immune cell types demonstrating a significant alteration in HPV16-PsVs uptake due to the presence of SP-A (Figure 2). Although not as pronounced as seen for HPV16, there was a significant reduction in the viral particle uptake in HaCaT cells for HPV types 18, 31 and 45 in the presence of SP-A (Figure 4). Interestingly, the corresponding MFI for all tested HPV types (i.e., 16, 18, 31, 45, 52 and 58) was significantly decreased in HaCaT cells. The viral particle uptake into RAW264.7 was increased for all tested HPV types, with HPV31 displaying the greatest increase in uptake compared to other types and HPV45 displaying the highest MFI (Figure 4).

As opposed to HPV16-PsVs, the particle uptake into THP-1 monocytes was not increased for any of the other HPV types tested when preincubated with SP-A. In fact, a decrease in MFI was observed for HPV types 18 and 31, although this was not statistically significant. Particle uptake in the presence of SP-A in DC0 was also type-dependent and differed from HPV16-PsVs. A notable decrease in the uptake for HPV18 was observed, with a decrease in MFI for HPV types 31, 45, 52 and 58 (Figure 4).

### 2.4. SP-A Enhances the Lysosomal Delivery of HPV16-PsVs in Immune and Epithelial Cells

**Figure 5:**
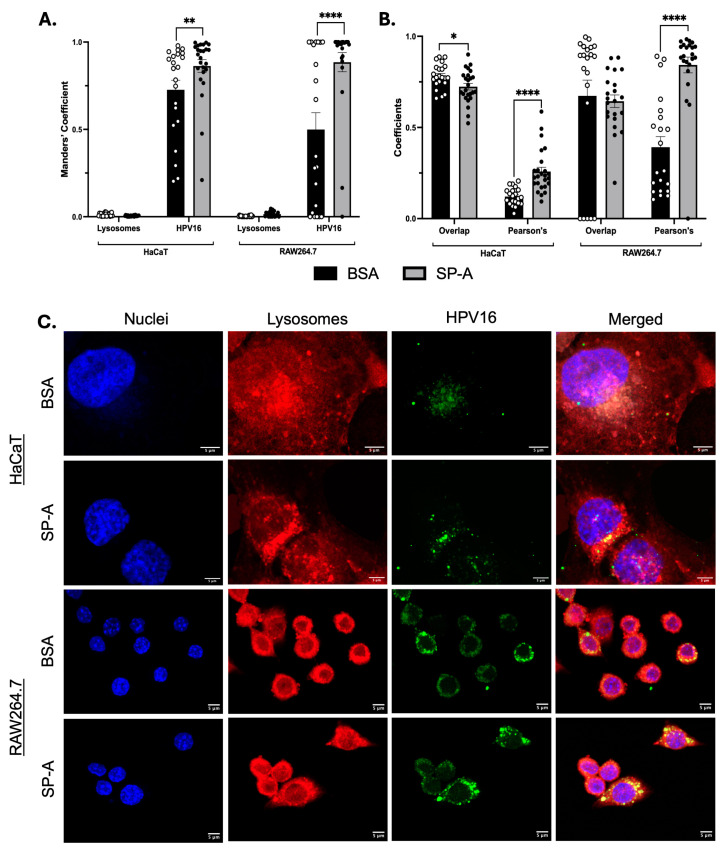
**Colocalisation characteristics of HPV16-PsVs and lysosomes for HaCaT and RAW264.7 at 8 h post-infection.** In total, 25 z-stack images were taken for each cell type and experimental condition using a Zeiss LSM 980 confocal microscope, and the resulting maximum intensity projections were used for the analysis. (**A**) Manders’ coefficients are indicated as Lysosomes (fraction of the lysosome signal overlapping with the HPV signal) and HPV16 (fraction of the HPV signal overlapping with the lysosome signal); 0.0 indicates no overlap and 1.0 indicates total overlap. (**B**) Overlap and Pearson’s coefficients are plotted. Each dot represents a single image, and the bars represent the mean and SEM. (**C**) Representative z-stacks of cells infected with AF488-HPV16-PsVs +/− SP-A for 8 h. Cells were stained with LysoTracker^TM^ Deep Red and Hoechst 33342 (blue). AF488-HPV16-PsVs were used and visualised in green. Statistical significance was determined by two-way ANOVA and Sidak’s multiple comparison tests. * = *p* < 0.05; ** = *p* < 0.01; **** = *p* < 0.0001; no symbol denotes “not significant”. Scale bar = 10 µm.

The colocalisation of AF488-HPV16-PsVs and lysosomes at 8 h post-infection was performed to assess the difference in the accumulation of HPV in these acidic compartments. We found that the fraction of the lysosome signal overlapping with the HPV signal was minimal, and no significant difference was seen for either HaCaT or RAW264.7 cells, regardless of the presence of SP-A (Figure 5A). The fraction of the HPV signal overlapping with the lysosome signal, however, was significantly higher in the SP-A groups for both HaCaT cells (mean value of 0.86 compared to BSA value of 0.73) and RAW264.7 cells (mean value of 0.88 compared to BSA value of 0.49) (Figure 5A).

When assessing the overlap coefficient, we found that the overlap of the HPV signal with the lysosome signal was slightly lower for the SP-A group than for the BSA control group in HaCaT but not RAW264.7 cells (Figure 5B). In addition, the Pearson’s coefficient was found to be significantly higher in the SP-A group for both HaCaT cells (mean value of 0.26 compared to BSA value of 0.12) and RAW264.7 cells (mean value of 0.84 compared to BSA value of 0.39) (Figure 5B). Taken together, these results indicate that the overall colocalisation pattern was similar in HaCaT and RAW264.7 cells, but the specific intensity correlation between HPV and lysosomes was increased in the presence of SP-A, indicating changes in their interaction dynamics or biological processes.

The occurrence of HPV and localisation with lysosomes for these cell lines in the absence or presence of SP-A is illustrated in Figure 5C. A qualitative analysis of the images for each experimental group showed an increase in the occurrence of the exocytosis of lysosomes (red vesicles that bud off from the cell membrane). Overall, a more intense LysoTracker signal and larger lysosomes in the presence of SP-A were observed, where these lysosomes were not necessarily carrying HPV particles.

### 2.5. SP-A Enhances the Immune Cell-Mediated Reduction in HPV16-PsVs Infection in Keratinocytes

**Figure 6:**
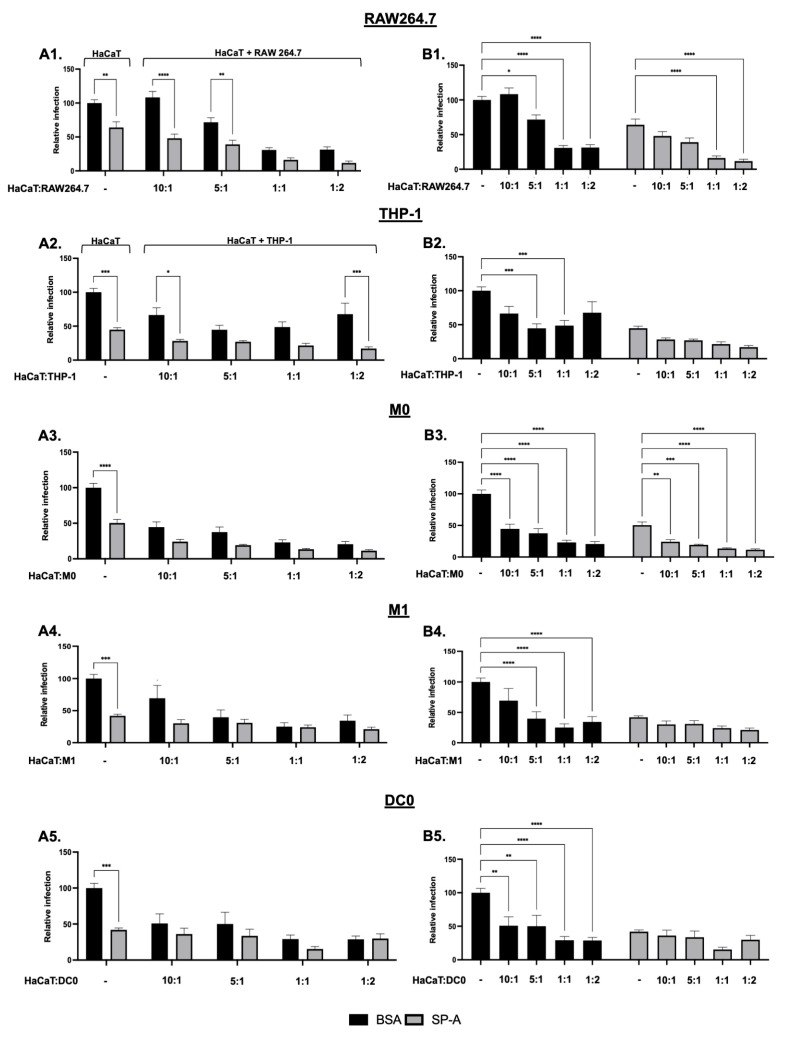
**HPV16-PsVs infection of HaCaT cells is decreased in the presence RAW264.7, THP-1, M0, M1 and DC0 cells and further dampened by preincubation with SP-A**. Briefly, 5000 HaCaT cells were plated with increasing numbers of RAW264.7 macrophages or THP-1-derived immune cells (depicted as ratios) the day prior to infection. HPV16-PsVs encapsidating the Gaussia luciferase reporter gene were preincubated with SP-A or BSA for 1 h at room temperature before being added to the cells. Depicted here are the infection levels of HaCaT cells after 48 h. (**A1**–**A5**) depict comparisons between BSA and SP-A for each culture. (**B1**–**B5**) depict comparisons between the different culture conditions within the same treatment group. Combinatorial analyses of three independent experiments are represented relative to the infection of the BSA control group (HaCaT only), which was set as 100%. Statistical significance was determined using two-way ANOVA with Sidak’s test for multiple comparisons. * = *p* < 0.05; ** = *p* < 0.01; *** = *p* < 0.001; **** = *p* < 0.0001; no symbol denotes “not significant”.

To assess the impact of immune cells on HaCaT cell infection by HPV16-PsVs preincubated with SP-A, a co-culture model was set up. SP-A preincubation led to a notable 40% reduction in single-cultured HaCaT infection after 48 h (Figure 6(A1)), consistent with the decreased initial HPV-PsVs uptake by HaCaT cells (Figure 2). Introducing RAW264.7 cells, which display minimal infectivity (i.e., luciferase activity) on their own [32], resulted in a significant dose-dependent reduction in HaCaT infection within the BSA control group (Figure 6(A1)). Adding SP-A-coated virions to the co-cultured cells led to an additive inhibitory effect on HPV16-PsVs infection, with the lowest infection levels observed in co-cultures with the highest macrophage numbers (Figure 6(B1)), suggesting a dose-dependent enhancement in the immune cell clearance of HPV16-PsVs, additive to SP-A’s inhibitory effect on HaCaT monocultures. Similar patterns were seen in THP-1 co-cultures. Introducing THP-1 cells decreased HPV16-PsVs’ infection of HaCaT cells in the BSA control (Figure 6(A2,B2)). The lowest infection levels were found with SP-A and a HaCaT:THP-1 ratio of 1:2, although they were not significantly lower than those of HaCaT monocultures infected with SP-A-coated HPV16-PsVs (Figure 6(B2)). M0 cells also displayed a dose-dependent reduction in infection in the BSA group (Figure 6(B3)), with SP-A further decreasing infection across co-culture conditions, albeit insignificantly (Figure 6(A3)). While the initial HPV16-PsVs uptake by M0 cells was not significantly changed by SP-A (Figure 2), M0 clearance appeared modulated by the presence of SP-A. HPV16-PsVs infection was significantly reduced by M1 cells in the BSA group in a dose-dependent manner (Figure 6(B4)). However, SP-A preincubation had less of an impact on infection with M1 macrophages compared to other immune cells (Figure 6(A4)). In DC0, HPV16-PsVs infection was similarly reduced (Figure 6(A5,B5)). Although the initial HPV16-PsVs uptake increased significantly in DC0 with SP-A (Figure 2), SP-A marginally decreased overall HaCaT infection when co-cultured with DC0 (Figure 6(A5,B5)). Although the presence of all immune cell types led to the decreased HPV infection of HaCaT cells to varying degrees, only M0 cells led to a further decreased infection in the presence of SP-A, which was statistically significant. While the mechanism behind this observation was not further investigated, one could speculate that specific M0–HaCaT cell interactions in the presence of SP-A (either via direct cell–cell contact, via specific cell surface molecule interactions or indirectly via the secretion of specific immunomodulatory molecules) prevented HPV infection (which was not as pronounced in the other immune cells tested). Moreover, SP-A may specifically enhance HPV’s intracellular processing, leading to more efficient degradation and reduced viral infectivity in M0 cells compared to other tested immune cells.

### 2.6. SP-A Contributes to the Immune Cell-Mediated Reduction in Infection by Multiple HPV-PsVs Types in HaCaT Cells

**Figure 7:**
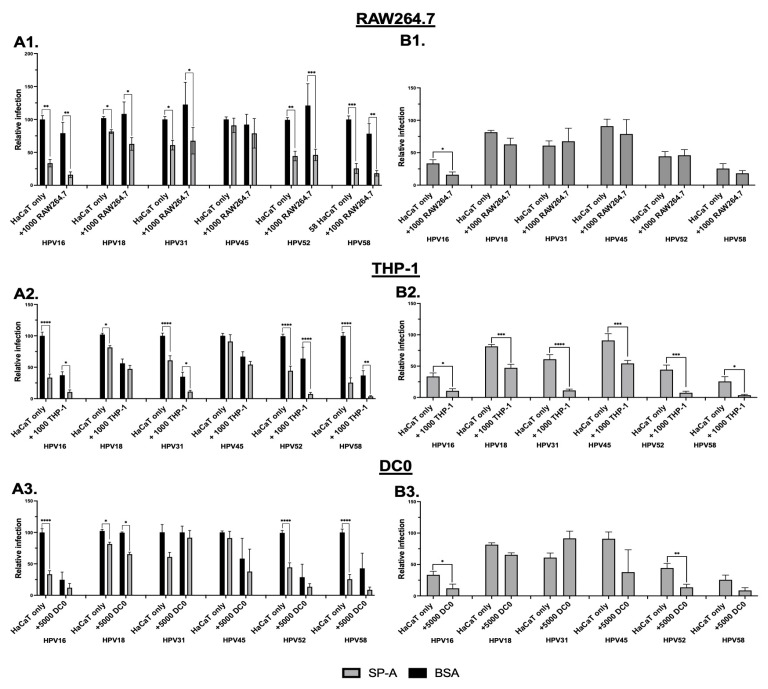
**Infection levels of HaCaT cells by selected oncogenic HPV types is dampened in the presence of SP-A and RAW264.7, THP-1 and DC0 cells**. Briefly, 5000 HaCaT cells were seeded with 1000 RAW264.7 cells, 1000 THP-1 cells or 5000 DC0 the day prior. HPV-PsVs types 16, 18, 31, 45, 52 and 58 encapsidating the Gaussia luciferase reporter gene were preincubated with SP-A or BSA for 1 h at room temperature before being added to the co-cultures. Depicted here are the infection levels at 72 h post infection, where (**A1**–**A3**) depicts comparisons between BSA and SP-A for each culture and (**B1**–**B3**) depicts comparisons between HaCaT monocultures and the addition of the respective immune cells in the SP-A group. Combinatorial analyses of three independent experiments are represented. Statistical significance was determined using two-way ANOVA with Sidak’s test for multiple comparisons. * = *p* < 0.05; ** = *p* < 0.01; *** = *p* < 0.001; **** = *p* < 0.0001; no symbol denotes “not significant”.

The infection of HaCaT cells by other oncogenic HPV-PsVs types in the presence of immune cells and SP-A was also assessed. RAW264.7, THP-1 and DC0 were chosen for this analysis. The highest HaCaT:immune cell ratio that showed a significant reduction in infection for HPV16-PsVs was chosen. This was 5:1 for RAW264.7 and THP-1 cells and 1:1 for DC0. SP-A reduced the infection of the HaCaT monocultures by the different HPV-PsVs types to varying degrees. Infection with HPV-PsVs types 18, 31, 52 and 58 was significantly reduced, comparable to what has been established for HPV16-PsVs (Figure 7(A1–A3)). The addition of RAW264.7 did not alter infection levels in the presence of SP-A for any HPV-PsVs type other than HPV16-PsVs (Figure 7(B1). The addition of THP1 and DC0 cells led to significant further changes in infection in the presence of SP-A for all types (THP-1 cells) and types 16 and 52 (DC0) (Figure 7(B2,B3)). Interestingly, the infection of HaCaT cells with HPV31-PsVs was increased in the DC0 co-cultures for the SP-A group, although not significantly (Figure 7(B3)).

### 2.7. SP-A Enhances the Cytokine Response to HPV16-PsVs by THP-1 Monocytes and THP-1-Derived DC0

**Figure 8:**
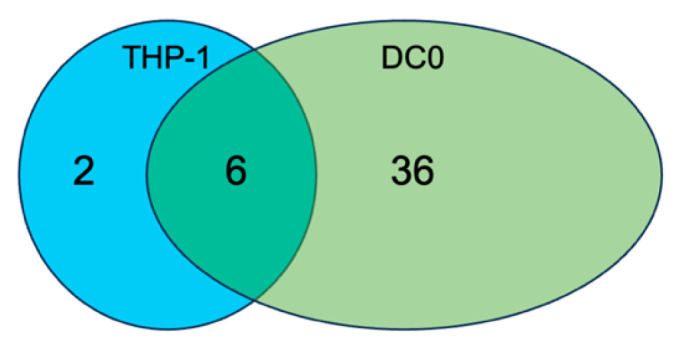
**Number of immune modulators differentially expressed in THP-1 and DC0 cells in the SP-A:HPV16-PsVs group compared to the untreated control at 24 hpi**.

Our study sought to investigate the impact of HPV16-PsVs infection on immune cell cytokine, chemokine and growth factor profiles, particularly in the presence of SP-A. We selected undifferentiated THP-1 monocytes and immature DCs for our immune modulator response towards HPV16-PsVs pre-incubated with or without SP-A due to their high phagocytic activity (Figure 2) and their ability to directly reduce HPV16-PsVs infection in HaCaT cells (Figure 7). The analysis of the Proteome Profiler^TM^ Human XL Cytokine Array included immune modulators that showed at least a 0.5-fold change in the expression (i.e., 50% reduction or increase in expression) of the SP-A:HPV16-PsVs group relative to the untreated control (Tables S1 and S2). DC0 cells exhibited a significantly higher degree of differential expression compared to THP-1 cells, with 36 unique immune modulators, compared to 2 in THP-1 cells. Six immune modulators were common to both cell types (Figure 8). This indicates a more pronounced response in DC0 cells when exposed to SP-A:HPV16-PsVs.

**Figure 9:**
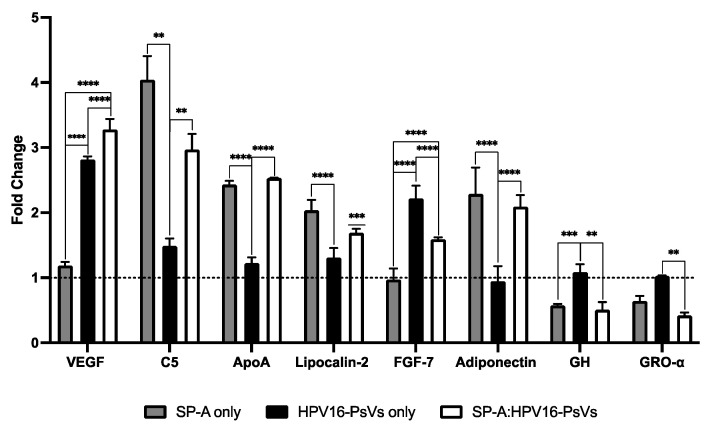
**Cytokines, chemokines and growth factors differentially expressed in the SP-A:HPV16-PsVs group compared to the untreated control in THP-1 cells at 24 hpi**. Data are represented as the mean fold-change relative to the untreated control group, which was set as 1 (dotted line). Statistical significance was determined using two-way ANOVA with Tukey’s test for multiple comparisons. ** = *p* < 0.001; *** = *p* < 0.001; **** = *p* < 0.0001; no symbol denotes “not significant”.

In THP-1 monocytes, most differentially expressed proteins were influenced by the presence of SP-A, regardless of HPV16-PsVs’ presence (Figure 9). VEGF, Complement component 5 (C5), Apoliprotein A (ApoA), Lipocalin-2, FGF-7 and Adiponectin were upregulated in the presence of SP-A, HPV16-PsVs and/or SP-A:HPV16-PsVs compared to the untreated control. Conversely, growth hormone (GH) and GRO-α were downregulated in the SP-A:HPV16-PsVs group compared to both the untreated and HPV16-PsVs-only groups.

**Figure 10:**
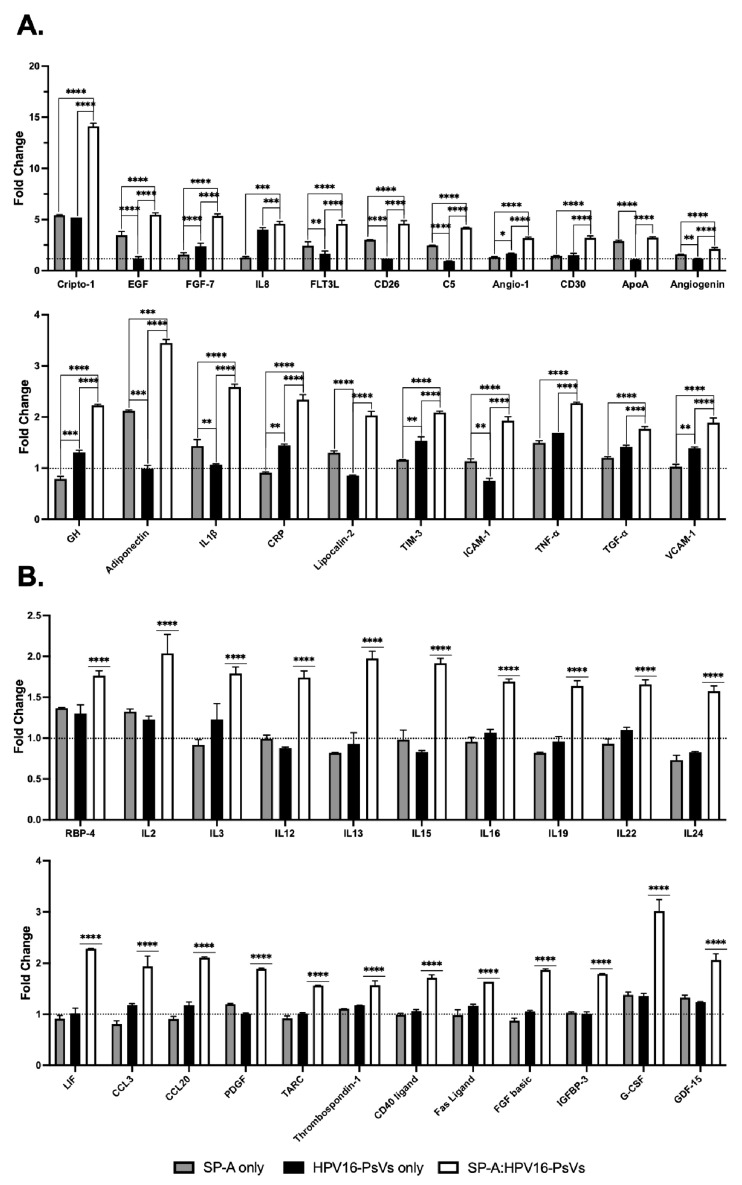
**Cytokines, chemokines and growth factors differentially expressed in the SP-A:HPV16-PsVs group compared to the untreated control in DC0 at 24 hpi**. Data are represented as the mean fold-change relative to the untreated control group, which was set as 1 (dotted line). (**A**) Immune modulators differentially expressed in SP-A alone and/or HPV16-PsVs alone and further modified by HPV16-PsVs in the presence of SP-A. (**B**) Immune modulators upregulated only in the dual presence of SP-A and HPV16-PsVs. Statistical significance was determined using two-way ANOVA with Tukey’s test for multiple comparisons. * = *p* < 0.05; ** = *p* < 0.001; *** = *p* < 0.001; **** = *p* < 0.0001; no symbol denotes “not significant”.

In contrast to the THP-1 cyctokine expression profile, which only showed a limited response to SP-A, DC0 cells displayed a substantial number of differentially expressed cytokines and growth factors. Immune modulators such as Cripto-1, EGF, FGF-7, IL-8, FLT3L and others were significantly upregulated in the presence of SP-A alone and/or HPV16-PsVs alone, with further modification observed in the SP-A:HPV16-PsVs group (Figure 10A). Additionally, several immune modulators, including LIF, CCL3, CCL20, PDGF, TARC, Thrombospondin-1 and others, were upregulated only in the dual presence of SP-A and HPV16-PsVs (Figure 10B).

**Figure 11:**
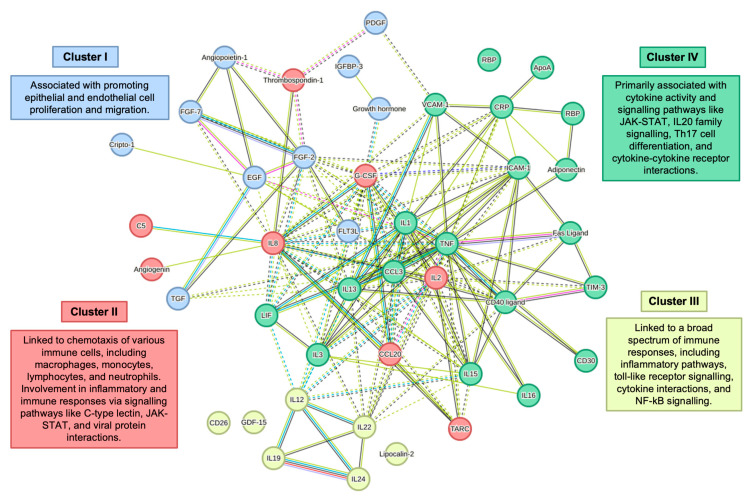
**STRING Protein–Protein Interaction Cluster Network for immune modulators upregulated in DC0 cells in the SP-A:HPV16-PsVs group**. This network map illustrates predicted functional interactions among the upregulated cytokines, chemokines and growth factors identified in DC0 when exposed to HPV16-PsVs and SP-A compared to untreated cells, as analysed using the STRING database. Nodes represent individual molecules; lines connecting nodes indicate known or predicted interactions. Proteins were grouped as indicated by the colour of the nodes, using kmeans clustering. Predicted interactions were determined as follows: from curated databases, experimentally determined, textmining, co-expression and protein homology. Solid lines represent interactions within clusters, and dotted lines connect proteins between clusters. Predicted protein–protein interactions were set to a high confidence threshold score.

Using the STRING database, we identified complex protein–protein interactions among the upregulated cytokines, chemokines and growth factors in DC0 cells exposed to SP-A:HPV16-PsVs (Figure 11). The analysis clustered immune modulators into four groups with distinct functional enrichments. Cluster I was associated with promoting epithelial and endothelial cell proliferation and migration. Cluster II was linked to the chemotaxis of immune cells, involving pathways such as C-type lectin, JAK-STAT and viral protein interactions. Cluster III included broad-spectrum immune responses, including toll-like receptor signalling, NF-κB signalling and cytokine interactions. Cluster IV was primarily associated with cytokine activity and signalling pathways such as JAK-STAT, IL20 family signalling, Th17 cell differentiation and cytokine–cytokine receptor interactions.

## 3. Discussion

HPV infection poses a significant global health challenge, especially in LMICs, where healthcare access and vaccine availability are limited. Despite the success of prophylactic vaccines against the most common oncogenic HPV types, these vaccines do not cover all oncogenic strains, and their efficacy is limited in women already infected with high-risk HPV types. This gap underscores the urgent need for alternative interventions to target a broader spectrum of HPV infections, particularly in regions with a high cervical cancer incidence.

Our research identified SP-A as a novel molecule capable of recognising HPV16 pseudovirions (HPV16-PsVs) and reducing infection in a murine cervicovaginal challenge model [32]. Building on these findings, we assessed SP-A’s suitability as a broad-spectrum HPV-targeting molecule and its impact on innate immune responses. Using cell-based techniques, we demonstrated that SP-A can agglutinate and opsonise multiple oncogenic HPV-PsVs types, although with varying potency, enhancing their uptake and clearance by selected immune cells such as murine macrophages (RAW264.7) and THP-1 human-derived monocytes and immature dendritic cells while not substantially influencing other tested immune cell populations. Selective SP-A mediated opsonisation and subsequent uptake into macrophages have also been observed for bacterial pathogens like *Mycobacterium tuberculosis*, *Escherichia coli*, *Streptococcus pneumoniae*, *Staphylococcus aureus* and *Streptococcus pneumoniae* [36,37,38]. SP-A has also been shown to improve phagocytosis and clearance by neutrophils [39]. Differences in HPV type susceptibility to SP-A may be due to their distinct capsid proteins and surface glycosylation profiles, which may affect the virions’ interaction with SP-A; however, the HPV surface only displays limited glycoproteins [40], and further research into these structural elements and interactions is crucial for developing therapeutic targets against HPV infections.

The selected cell models used here (i.e., murine RAW264.7 macrophages and THP-1-derived immune cells) are widely accepted in studying phagocytosis and immune responses. While RAW264.7 cells provide a robust model and are highly efficient at phagocytosis, THP-1 cells are more versatile, as they can differentiate into various immune cell types with distinct functional profiles, demonstrating significant phagocytic activity that varies with their differentiation state. Significant differences in the protein expression profiles have been observed between THP-1 and RAW264.7 cells when challenged with the same stimuli, highlighting the differences in their response [41,42]. Consequently, THP-1-derived macrophages are preferred for studying human-specific pathogen interactions, while RAW264.7 cells are used for general studies of macrophage function and murine model system comparisons. The differences in the response to HPV (in the presence and absence of SP-A) between these cell lines, as described in our study, are therefore not surprising. While acknowledging that engineered cell lines and in vitro systems may not perfectly replicate the entirety of native immune cell heterogeneity, they remain indispensable tools in uncovering fundamental immune mechanisms and providing preliminary insights into immune responses in controlled settings.

Exogenous supplementation with SP-A not only enhanced the recognition and uptake of HPV-PsVs by immune cells but also simultaneously reduced viral uptake and infection in HaCaT human keratinocytes, probably due to agglutination and viral cluster formation. The internalisation pathways appear to differ between immune cells and keratinocytes with immune cells primarily utilising phagocytosis, while keratinocytes employ receptor-mediated endocytosis. SP-A opsonisation thus has distinct effects on these pathways, reflecting the different mechanisms of pathogen uptake in these cell types. Similar agglutination effects of SP-A as seen here have been documented for other pathogens, such as influenza virus, where SP-A facilitated viral aggregation and subsequent clearance by macrophages [43,44,45]. We then further tested the effect of SP-A on various immune cell types and their effectiveness in reducing downstream HPV-PsVs infection of HaCaT cells. While RAW264.7 macrophages, THP-1 monocytes and M0 showed sensitivity to SP-A mediated HPV-PsVs clearance, resulting in decreased infection of HaCaT cells, M1 macrophages and DC0 substantially reduced HaCaT cell infection, relatively independently of SP-A. These findings underscore the distinct roles of various immune cells in combating HPV-PsVs infection, both with and without the influence of SP-A. However, the dual function of SP-A regarding HPV infection—facilitating the clearance by selected immune cells and preventing keratinocyte infection—suggests significant therapeutic potential.

Intracellular trafficking studies revealed that SP-A enhances the lysosomal delivery of HPV16-PsVs in both keratinocytes and macrophages. This increased lysosomal accumulation suggests that SP-A promotes the degradation of HPV particles within these acidic compartments, potentially reducing viral infectivity. Confocal microscopy confirmed that SP-A increases the association between HPV16-PsVs and lysosomes, highlighting its role in modulating intracellular trafficking pathways. SP-A has previously been shown to be rapidly endocytosed by a receptor-mediated process through clathrin-coated pits and is trafficked through the endolysosomal pathway in human macrophages [46]. Furthermore, SP-A has been shown to enhance the lysosomal delivery of *E. coli* in RAW264.7 cells by regulating Rab7 expression [47]. However, it was previously unknown whether SP-A also plays a role in the intracellular trafficking of HPV in epithelial cells and/or immune cells.

In addition to enhancing viral clearance, we showed that SP-A modulated cytokine and chemokine production in response to HPV16-PsVs. THP-1 monocytes and DC0 cells exhibited a significant inflammatory response when exposed to SP-A-opsonised HPV16-PsVs. This was seen by the upregulation of multiple proinflammatory cytokines and immune modulators, with DC0 displaying a more pronounced and multi-faceted response compared to THP-1 cells. This proinflammatory response is crucial for effective immune defense, indicating SP-A’s role in enhancing immune cell recruitment and activation [48]. The upregulation of specific cytokines and chemokines, such as C5/C5a, Apolipoprotein A-I, Lipocalin-2, Adiponectin, VEGF and FGF-7, underscores SP-A’s ability to modulate immune responses favourably towards pathogen clearance and tissue repair [49,50,51,52,53,54]. STRING analysis further elucidated the complex network of interactions among these cytokines, chemokines and growth factors, revealing their involvement in critical pathways like JAK-STAT signalling, NF-κB signalling and toll-like receptor signalling. These pathways are essential for orchestrating effective immune responses, highlighting SP-A’s potential to bridge innate and adaptive immunity [48]. SP-A’s ability to modulate cytokine profiles has been previously documented in response to other respiratory pathogens, such as respiratory syncytial virus (RSV), where SP-A enhanced the production of inflammatory mediators, promoting pathogen clearance [33,55].

**Figure 12:**
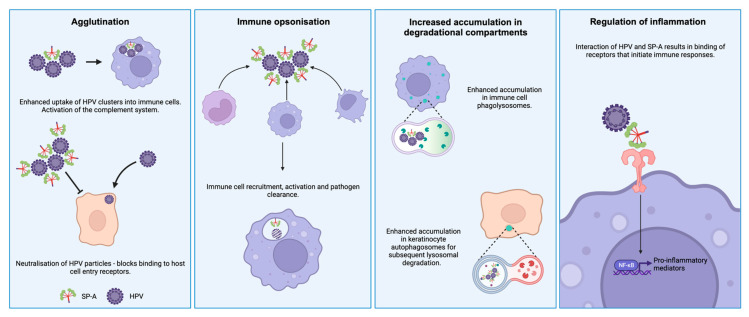
**Proposed mechanisms of SP-A immunomodulation in the context of HPV infection.** SP-A binds and agglutinates HPV, resulting in an increased uptake of larger HPV clusters into immune cells and a decreased uptake into keratinocytes as a result of hindering HPV binding to cell surface receptors. SP-A opsonises HPV, resulting in immune cell recruitment, activation and pathogen clearance. SP-A affects the intracellular trafficking of HPV by enhancing the accumulation of viral particles in the phagolysosomes of immune cells and redirecting HPV endocytosis to autophagosomes and subsequent lysosomal degradation. SP-A opsonisation of HPV results in SP-A binding to immune cell receptors and the subsequent initiation of a proinflammatory response. Image made with BioRender.com.

Given SP-A’s broad-spectrum activity and ability to modulate immune responses (Figure 12), it holds promise as a component in alternative prophylactic interventions. The development of SP-A-based topical microbicides could provide a cost-effective and easily accessible method for preventing HPV infections, particularly in LMICs, where traditional vaccination programs face significant barriers. Future research should focus on identifying specific SP-A interaction sites on HPV, developing small peptide inhibitors based on SP-A and evaluating their efficacy in clinical settings.

## 4. Materials and Methods

### 4.1. Cell Culture

The virus packaging cell line HEK-293TT [56], the human epithelial HPV18-positive cervical adenocarcinoma cell line HeLa, the spontaneously immortalised keratinocyte cell line HaCaT and the murine leukemic macrophage cell line RAW264.7 were grown and maintained in DMEM (Lonza, Basel, Switzerland) supplemented with 10% heat-inactivated foetal calf serum (FCS) (Biochrom, Cambridge, UK), 100 U/mL penicillin and 100 μg/mL streptomycin. The spontaneously immortalised human keratinocyte cell line NIKS [57] was grown and maintained in F medium (3:1 (*v*/*v*) Ham’s F-12 K medium: DMEM; 5% heat-inactivated FCS; 0.4 µg/mL hydrocortisone (Merck, Kenilworth, NJ, USA); 5 µg/mL insulin (Sigma, St. Louis, MO, USA); 8.4 ng/mL cholera toxin (Sigma); 10 ng/mL recombinant human Epidermal Growth Factor (Life Technologies, Carlsbad, CA, USA); 24 µg/mL adenine (Sigma); 100 U/mL penicillin and 100 μg/mL streptomycin). The spontaneously immortalised human monocytic-like cell line THP-1—like all cells derived from this cell line—was grown and maintained in RPMI (Lonza) supplemented with 10% heat-inactivated FCS, 100 U/mL penicillin and 100 μg/mL streptomycin. All cells were grown at 37 °C in a 5% CO_2_/95% air humidified atmosphere.

### 4.2. Human Innate Immune Cell Panel (ICP)

The differentiation of THP-1 to steady state macrophages (M0) was adapted from published protocols [58,59]. Briefly, THP-1 cells were stimulated at a density of 1.0 × 10^6^ cells/mL with 10 ng/μL phorbol 12-myristate 13-acetate (PMA, Sigma) for 24 h, washed and rested in fresh complete RPMI for 48 h before further usage. The differentiation of M0 macrophages to M1, M2a or M2c populations was adapted from [60,61]. Briefly, M0 macrophages that had been resting post-PMA exposure for 24 h were exposed to 100 ng/mL LPS (Sigma) and 20 ng/mL IFN-γ (Peprotech, Cranbury, NJ, USA) (M1), 20 ng/mL IL-4 (Thermo Fisher Scientific, Waltham, MA, USA) (M2a) or 20 ng/mL IL-10 (Peprotech) (M2c) for 24 h in RPMI containing 1% heat-inactivated FCS. The differentiation of THP-1 cells to immature DCs (DC0) was adapted from [62,63]. Briefly, THP-1 cells were stimulated at a density of 0.5 × 10^6^ cells/mL with 10 ng/mL recombinant human Granulocyte-Macrophage Colony-Stimulating Factor (rhGM-CSF, Thermo Fisher Scientific) and 20 ng/mL IL-4 (Thermo Fisher Scientific) in RPMI containing 2% heat-inactivated FCS for 6 days, replenishing rhGM-CSF and IL-4 in fresh media on day 3. The innate immune cell populations were characterised by multi-colour flow cytometry using the following fluorochrome-conjugated antibodies: HLA-DR PE clone L243 (BioLegend, San Diego, CA, USA), CD11c Brilliant Violet (BV) 650 clone B-ly6 (BD Biosciences, Franklin Lakes, NJ, USA), CD86 FITC clone BU63 (BioLegend), CD169 BV605 clone 7-239 (BioLegend), CD206 PE-Cy5 clone 15-2 (BioLegend) and DC-SIGN BV421 clone 9E9A8 (BioLegend). The cells were stained in staining buffer (5% *(w/v)* BSA (Sigma) in PBS) for 30 min at 4 °C (dark), washed and resuspended in FACS FIX (1% (*v*/*v*) formaldehyde (Sigma) in PBS) for acquisition using the BD LSRFortessa. The data were analysed using FlowJo software (v10.7.2). Innate immune cells were characterised by their relative expression of cell surface receptors, and t-SNE analysis revealed distinct clusters corresponding to different immune cell subsets, indicating unique expression profiles and potential heterogeneity within the THP-1 cell line (Figures S3 and S4).

### 4.3. HPV Pseudovirus Preparation and Labelling

HPV pseudovirions (HPV-PsVs) encapsidating the secreted Gaussia luciferase reporter gene plasmid pCMV-GLuc2 (New England Biolabs, Ipswich, MA, USA) were produced in HEK-293TT cells by co-transfection with the plasmids pXULL (for HPV type 16), pV18cap (for HPV type 18), p31sheLL (for HPV type 31), p45sheLL (for HPV type 45), p52 (for HPV type 52) and p58sheLL (for HPV type 58), which encode codon-optimised type-specific L1 and L2, following published purification procedures and quantification and quality controls [32,64,65]. All L1/L2-encoding plasmids were kindly provided by John Schiller (National Institutes of Health, Bethesda, MD, USA). Where indicated, the virions were labelled with Alexa Fluor 488 succinimidyl ester (AF488; Life Technologies) before purification by CsCl density gradient centrifugation [32,64,65]. Unless otherwise stated, the cells were infected at an HPV-PsVs concentration of 2–4 pg/cell.

### 4.4. Purification of Native Human SP-A

Human SP-A was purified from bronchoalveolar lavage (BAL) fluid from patients with alveolar proteinosis using a butanol extraction method, as previously described [55].

BAL was collected with informed consent and ethical approval (Royal Brompton and Harefield Research Ethics Committee NRES10/H0504/9). The endotoxin levels in the SP-A preparation were measured using a Limulus Amoebocyte Lysate (LAL) assay (Lonza), and levels < 1 pg/μg were acceptable. The purified proteins were filtered (0.22 μm) and stored at −20 °C until use.

### 4.5. Experiments with SP-A-Opsonised HPV-PsVs

Unless otherwise stated, the HPV-PsVs were opsonised with SP-A or the control protein BSA by preincubating the viral particles with SP-A (or BSA) at room temperature for 1 h in 2 mL low-protein-binding Eppendorf tubes at a 1:10 PsVs-to-SP-A (or BSA) ratio, in the presence of 5 mM sterile filtered CaCl_2_, before usage [32].

#### 4.5.1. HPV-PsVs Internalisation Assays

To assess viral binding and internalisation by flow cytometry, the cells were plated in triplicate in 1 mL complete media in 12-well plates at a density of 100,000 cells/mL and grown overnight. Opsonised AF488-labelled HPV-PsVs were added to the cells at a final viral density of approximately 4 pg/cell for 1 h at 37 °C. The cells were rinsed with PBS and harvested with 0.025% trypsin/0.01% EDTA in PBS to remove surface-bound virions, thereby allowing for the detection of internalised AF488-labelled viral particles by flow cytometry [32]. The cells were washed in FACS WASH solution (0.5% BSA in PBS) and fixed with FACS FIX. The detection of AF488-positive cells was performed using a BD LSRFortessa^TM^ Cell Analyser using the BD FACSDiva v9.0 software in the AF488 channel. Acquisition parameters were set up using an unstained negative control and stained positive control. FlowJo^TM^ software (v10.7.2; BD Biosciences) was used for post-acquisition analysis. Given that SP-A was hypothesised to aggregate viral particles, we evaluated the MFI of the HPV-positive cells. This additional measure was analysed to provide a more nuanced understanding of the internalisation intensity, offering insight into the extent of viral particle uptake per cell beyond simple presence or absence.

#### 4.5.2. HPV-PsVs Infection Assays

A direct cell-to-cell co-culture system consisting of HaCaT cells as the HPV-infectible epithelial cell line and RAW264.7-, THP-1- and THP-1-derived immune cells was set up in 96-well plates, unless otherwise stated. Briefly, HaCaT cells were plated at a density of 5000 cells per well, together with immune cells at approximate epithelial-cell-to-immune-cell ratios of 10:1, 5:1, 1:1 and 1:2, respectively. The cells were grown overnight before subsequent infection with 0.1 μg HPV-PsVs opsonised with 1 μg SP-A or the BSA control per well. Infection was monitored by Gaussia luciferase activity measured in the cell supernatant 24 h, 48 and 72 h post-infection by adding 50 μL of the freshly prepared GLuc Assay Solution (Gaussia Luciferase Assay Kit, New England Biolabs) to 5 μL of the cell supernatant. Luminescence was measured with a 2 s lag time over a 10 s integration time using a GloMax^®^ Explorer Multimode Microplate Reader (Promega, Madison, WI, USA).

#### 4.5.3. Immunofluorescence and Confocal Microscopy

To investigate the uptake of opsonised HPV16-PsVs, HaCaT and RAW264.7 cells were grown overnight on sterile square coverslips (22 × 22 mm, Marienfield Superior) and subjected to the viral particles for 3 h. The cells were washed with warm PBS and fixed with 2% (*v*/*v*) paraformaldehyde for 5 min at 37 °C before being washed again and stained with 5 μg/mL Hoechst 33342 (Thermo Fisher Scientific) in PBS for 5 min at room temperature. To assess lysosomal accumulation at 8 h post-infection, opsonised virions were added to the cells for 7 h, followed by live staining with LysoTracker™ Deep Red (Thermo Fisher Scientific) in warm DMEM for 1 h at 37 °C at a concentration of 50 nM for HaCaT cells and 75 nM for RAW264.7 cells. The cells were gently washed with warm PBS before fixing and staining with Hoechst 33342.

Imaging was performed on a Zeiss LSM 980 with an Airyscan 2 confocal laser scanning microscope (Carl Zeiss, Oberkochen, Germany). Z-stack intervals were set at 1 μm, and a total of five slices were acquired. Fiji (ImageJ2, v2.14.0/1.54f) was used to perform the particle and colocalisation analyses on the maximum intensity projections produced from the z-stacks. For particle analyses, the lower upper threshold values for the green channel for both HaCaT and RAW264.7 cells (3900–65535 and 2500–65535, respectively) were manually set and kept constant. For colocalisation analyses, the lower and upper threshold values for the green channel for both HaCaT and RAW264.7 cells (1250–65535 and 811–65535, respectively) and the red channel (2250–65535 and 5010–65535, respectively) were manually set and kept constant to separate the signal from the background using the single-stained controls. The “JACoP” (Just Another Colocalisation) plugin (v2.1.4) was used to assess the colocalisation of HPV in lysosomes. The overlap coefficient, Pearson’s correlation coefficient and Manders’ coefficient were recorded to quantify the degree of colocalisation between the red and green channels (lysosomes and HPV, respectively) [66].

To correct for chromatic shifts when capturing the channels, we used the calibration MultiSpecTM bead sample (Zeiss). Images of the beads were captured using the same acquisition settings used for the experiment. The Align.ijm macro script was run to align the green, red and blue channels of the beads. The calculated transformation was saved as a .csv file to be later applied to all experimental images.

#### 4.5.4. Cytokine Assays

The levels of immune modulatory proteins were assessed for THP-1 monocytes and DC0 using the Proteome Profiler^TM^ Human XL Cytokine Array Kit (R&D Systems, Minneapolis, MN, USA). In total, 250,000 THP-1 and DC0 cells were plated (in parallel) in 1 mL RPMI in 12-well plates and cultured overnight, followed by the addition of HPV16-PsVs preincubated with BSA, HPV16-PsVs preincubated with SP-A or SP-A alone, or they were left untreated. The cell supernatants were harvested 24 h post-infection and processed according to the manufacturer’s instructions. The membrane images were analysed using QuickSpots v25.6.0.3 (Ideal Eyes Systems, Bountiful, UT, USA), which calculates the pixel density at each site. The fold changes in cytokine expression were calculated and reported as changes relative to the pixel densities for the untreated membrane.

To explore potential protein interactions from the cytokine expression data, the Search Tool for the Retrieval of Interacting Genes/Proteins (STRING, v12.0) was used. 

### 4.6. Statistical Analysis

One-way analysis of variance (ANOVA) was employed to assess the significance of differences among multiple groups. To further examine pairwise comparisons between individual groups, Dunnett’s multiple comparison test was applied. Additionally, a two-way ANOVA was performed to investigate the influence of two independent variables on the dependent variable. To determine significant differences in pairwise comparisons within the two-way ANOVA, the Sidak multiple comparison test was employed. All calculations were performed with Graph Pad Prism v9.

## 5. Conclusions

Our study highlights SP-A’s significant impact on HPV prevention strategies by enhancing viral immune recognition and modulating immune responses. SP-A offers a promising avenue for broad-spectrum protection against diverse HPV types and potentially other sexually transmitted infections. Further research into SP-A’s mechanisms and therapeutic applications could lead to innovative and accessible prevention strategies.

## Data Availability

The data presented in this study are available on request from the corresponding author.

## Supplementary Materials

The following supporting information can be downloaded at: https://www.mdpi.com/article/10.3390/ijms25147712/s1.

## References

[1] Bosch F.X., Manos M.M., Muñoz N., Sherman M., Jansen A.M., Peto J., Schiffman M.H., Moreno V., Kurman R., Shan K.V. (1995). Prevalence of human papillomavirus in cervical cancer: A worldwide perspective. JNCI J. Natl. Cancer Inst..

[2] de Martel C., Georges D., Bray F., Ferlay J., Clifford G.M. (2020). Global burden of cancer attributable to infections in 2018: A worldwide incidence analysis. Lancet Glob. Health.

[3] Plummer M., de Martel C., Vignat J., Ferlay J., Bray F., Franceschi S. (2016). Global burden of cancers attributable to infections in 2012: A synthetic analysis. Lancet Glob. Health.

[4] Muñoz N., Bosch F.X., De Sanjosé S., Herrero R., Castellsagué X., Shah K.V., Snijders P.J., Meijer C.J. (2003). Epidemiologic classification of human papillomavirus types associated with cervical cancer. N. Engl. J. Med..

[5] HPV and Cancer—NCI. https://www.cancer.gov/about-cancer/causes-prevention/risk/infectious-agents/hpv-and-cancer.

[6] Bruni L.A.G., Serrano B., Mena M., Collado J.J., Gómez D., Muñoz J., Bosch F.X., de Sanjosé S. (2023). Human Papillomavirus and Related Diseases in South Africa.

[7] De Vuyst H., Alemany L., Lacey C., Chibwesha C.J., Sahasrabuddhe V., Banura C., Denny L., Parham G.P. (2013). The burden of human papillomavirus infections and related diseases in sub-saharan Africa. Vaccine.

[8] Williamson A.L. (2015). The interaction between human immunodeficiency virus and human papillomaviruses in heterosexuals in Africa. J. Clin. Med..

[9] Sharma K., Machalek D.A., Toh Z.Q., Amenu D., Muchengeti M., Ndlovu A.K., Mremi A., Mchome B., Vallely A.J., Denny L. (2023). No woman left behind: Achieving cervical cancer elimination among women living with HIV. Lancet HIV.

[10] Denny L. (2015). Control of cancer of the cervix in low-and middle-income countries. Ann. Surg. Oncol..

[11] Amzat J., Kanmodi K.K., Aminu K., Egbedina E.A. (2023). School-based interventions on human papillomavirus in Africa: A systematic scoping review. Venereology.

[12] Milondzo T., Meyer J.C., Dochez C., Burnett R.J. (2022). Human papillomavirus vaccine hesitancy highly evident among caregivers of girls attending South African private schools. Vaccines.

[13] Tathiah N., Naidoo M., Moodley I. (2015). Human papillomavirus (HPV) vaccination of adolescents in the South African private health sector: Lessons from the HPV demonstration project in KwaZulu-Natal. S. Afr. Med. J..

[14] Ngcobo N., Burnett R., Cooper S., Wiysonge C. (2019). Human papillomavirus vaccination acceptance and hesitancy in South Africa: Research and policy agenda. S. Afr. Med. J..

[15] Ledibane T.D., Ledibane N.R., Matlala M. (2023). Performance of the school-based human papillomavirus vaccine uptake in Tshwane, South Africa. S. Afr. J. Infect. Dis..

[16] Carse S., Bergant M., Schafer G. (2021). Advances in Targeting HPV Infection as Potential Alternative Prophylactic Means. Int. J. Mol. Sci..

[17] Zhou C., Tuong Z.K., Frazer I.H. (2019). Papillomavirus immune evasion strategies target the infected cell and the local immune system. Front. Oncol..

[18] Roden R., Stern P.L. (2018). Opportunities and challenges for human papillomavirus vaccination in cancer. Nat. Rev. Cancer.

[19] Stanley M.A. (2012). Epithelial cell responses to infection with human papillomavirus. Clin. Microbiol. Rev..

[20] Narisawa-Saito M., Kiyono T. (2007). Basic mechanisms of high-risk human papillomavirus-induced carcinogenesis: Roles of E6 and E7 proteins. Cancer Sci..

[21] Li H., Ou X., Xiong J., Wang T. (2006). HPV16E7 mediates HADC chromatin repression and downregulation of MHC class I genes in HPV16 tumorigenic cells through interaction with an MHC class I promoter. Biochem. Biophys. Res. Commun..

[22] Park J.-S., Kim E.-J., Kwon H.-J., Hwang E.-S., Namkoong S.-E., Um S.-J. (2000). Inactivation of interferon regulatory factor-1 tumor suppressor protein by HPV E7 oncoprotein: Implication for the E7-mediated immune evasion mechanism in cervical carcinogenesis. J. Biol. Chem..

[23] Riera Romo M., Pérez-Martínez D., Castillo Ferrer C. (2016). Innate immunity in vertebrates: An overview. Immunology.

[24] Medzhitov R., Janeway C.A. (1997). Innate immunity: Impact on the adaptive immune response. Curr. Opin. Immunol..

[25] Marshall J.S., Warrington R., Watson W., Kim H.L. (2018). An introduction to immunology and immunopathology. Allergy Asthma Clin. Immunol..

[26] Turvey S.E., Broide D.H. (2010). Innate immunity. J. Allergy Clin. Immunol..

[27] Geissmann F., Manz M.G., Jung S., Sieweke M.H., Merad M., Ley K. (2010). Development of monocytes, macrophages, and dendritic cells. Science.

[28] Hume D.A., Ross I.L., Himes S.R., Sasmono R.T., Wells C.A., Ravasi T. (2002). The mononuclear phagocyte system revisited. J. Leukoc. Biol..

[29] Hirayama D., Iida T., Nakase H. (2017). The phagocytic function of macrophage-enforcing innate immunity and tissue homeostasis. Int. J. Mol. Sci..

[30] Pivarcsi A., Kemény L., Dobozy A. (2004). Innate immune functions of the keratinocytes. Acta Microbiol. Et Immunol. Hung..

[31] Yatim K.M., Lakkis F.G. (2015). A brief journey through the immune system. Clin. J. Am. Soc. Nephrol..

[32] Ujma S., Carse S., Chetty A., Horsnell W., Clark H., Madsen J., Mackay R.M., Watson A., Griffiths M., Katz A.A. (2019). Surfactant Protein A Impairs Genital HPV16 Pseudovirus Infection by Innate Immune Cell Activation in A Murine Model. Pathogens.

[33] Watson A., Sørensen G.L., Holmskov U., Whitwell H.J., Madsen J., Clark H. (2020). Generation of novel trimeric fragments of human SP-A and SP-D after recombinant soluble expression in *E. coli*. Immunobiology.

[34] Ujma S., Horsnell W.G., Katz A.A., Clark H.W., Schäfer G. (2017). Non-Pulmonary Immune Functions of Surfactant Proteins A and D. J. Innate Immun..

[35] Wright J.R. (2005). Immunoregulatory functions of surfactant proteins. Nat. Rev. Immunol..

[36] Gaynor C.D., Mccormack F.X., Voelker D.R., McGowan S.E., Schlesinger L.S. (1995). Pulmonary surfactant protein A mediates enhanced phagocytosis of *Mycobacterium tuberculosis* by a direct interaction with human macrophages. J. Immunol..

[37] Pikaar J.C., Voorhout W.F., van Golde L.M., Verhoef J., Van Strijp J.A., van Iwaarden J.F. (1995). Opsonic activities of surfactant proteins A and D in phagocytosis of gram-negative bacteria by alveolar macrophages. J. Infect. Dis..

[38] McNeely T.B., Coonrod J.D. (1993). Comparison of the opsonic activity of human surfactant protein A for Staphylococcus aureus and Streptococcus pneumoniae with rabbit and human macrophages. J. Infect. Dis..

[39] Hartshorn K.L., Crouch E., White M.R., Colamussi M.L., Kakkanatt A., Tauber B., Shepherd V., Sastry K.N. (1998). Pulmonary surfactant proteins A and D enhance neutrophil uptake of bacteria. Am. J. Physiol.-Lung Cell. Mol. Physiol..

[40] Mobini Kesheh M., Shavandi S., Azami J., Esghaei M., Keyvani H. (2023). Genetic diversity and bioinformatic analysis in the L1 gene of HPV genotypes 31, 33, and 58 circulating in women with normal cervical cytology. Infect. Agents Cancer.

[41] Li P., Hao Z., Wu J., Ma C., Xu Y., Li J., Lan R., Zhu B., Ren P., Fan D. (2021). Comparative proteomic analysis of polarized human THP-1 and mouse RAW264. 7 macrophages. Front. Immunol..

[42] Fitzgerald M.L., Moore K.J., Freeman M.W., Reed G.L. (2000). Lipopolysaccharide induces scavenger receptor A expression in mouse macrophages: A divergent response relative to human THP-1 monocyte/macrophages. J. Immunol..

[43] Kishore U., Greenhough T.J., Waters P., Shrive A.K., Ghai R., Kamran M.F., Bernal A.L., Reid K.B., Madan T., Chakraborty T. (2006). Surfactant proteins SP-A and SP-D: Structure, function and receptors. Mol. Immunol..

[44] Ghildyal R., Hartley C., Varrasso A., Meanger J., Voelker D.R., Anders E.M., Mills J. (1999). Surfactant protein A binds to the fusion glycoprotein of respiratory syncytial virus and neutralizes virion infectivity. J. Infect. Dis..

[45] Hartshorn K.L., White M.R., Shepherd V., Reid K., Jensenius J.C., Crouch E. (1997). Mechanisms of anti-influenza activity of surfactant proteins A and D: Comparison with serum collectins. Am. J. Physiol.-Lung Cell. Mol. Physiol..

[46] Crowther J.E., Schlesinger L.S. (2006). Endocytic pathway for surfactant protein A in human macrophages: Binding, clathrin-mediated uptake, and trafficking through the endolysosomal pathway. Am. J. Physiol.-Lung Cell. Mol. Physiol..

[47] Sender V., Moulakakis C., Stamme C. (2011). Pulmonary surfactant protein A enhances endolysosomal trafficking in alveolar macrophages through regulation of Rab7. J. Immunol..

[48] Watson A., Madsen J., Clark H.W. (2021). SP-A and SP-D: Dual functioning immune molecules with antiviral and immunomodulatory properties. Front. Immunol..

[49] Manthey H.D., Woodruff T.M., Taylor S.M., Monk P.N. (2009). Complement component 5a (c5a). Int. J. Biochem. Cell Biol..

[50] Yang P., Skiba N.P., Tewkesbury G.M., Treboschi V.M., Baciu P., Jaffe G.J. (2017). Complement-mediated regulation of apolipoprotein E in cultured human RPE cells. Investig. Ophthalmol. Vis. Sci..

[51] Flo T.H., Smith K.D., Sato S., Rodriguez D.J., Holmes M.A., Strong R.K., Akira S., Aderem A. (2004). Lipocalin 2 mediates an innate immune response to bacterial infection by sequestrating iron. Nature.

[52] Dikmen K., Bostanci H., Gobut H., Yavuz A., Alper M., Kerem M. (2018). Recombinant adiponectin inhibits inflammation processes via NF-kB pathway in acute pancreatitis. Bratisl. Med. J.-Bratisl. Lek. Listy.

[53] Powers C., McLeskey S., Wellstein A. (2000). Fibroblast growth factors, their receptors and signaling. Endocr.-Relat. Cancer.

[54] Wells A. (1999). EGF receptor. Int. J. Biochem. Cell Biol..

[55] Watson A., Kronqvist N., Spalluto C.M., Griffiths M., Staples K.J., Wilkinson T., Holmskov U., Sorensen G.L., Rising A., Johansson J. (2017). Novel expression of a functional trimeric fragment of human SP-A with efficacy in neutralisation of RSV. Immunobiology.

[56] Buck C.B., Pastrana D.V., Lowy D.R., Schiller J.T., Davy C., Doorbar J. (2006). Generation of HPV Pseudovirions Using Transfection and Their Use in Neutralization Assays. Human Papillomaviruses: Methods and Protocols.

[57] Allen-Hoffmann B.L., Schlosser S.J., Ivarie C.A., Sattler C.A., Meisner L.F., O’Connor S.L. (2000). Normal growth and differentiation in a spontaneously immortalized near-diploid human keratinocyte cell line, NIKS. J. Investig. Dermatol..

[58] Daigneault M., Preston J.A., Marriott H.M., Whyte M.K., Dockrell D.H. (2010). The identification of markers of macrophage differentiation in PMA-stimulated THP-1 cells and monocyte-derived macrophages. PLoS ONE.

[59] Aldo P.B., Craveiro V., Guller S., Mor G. (2013). Effect of culture conditions on the phenotype of THP-1 monocyte cell line. Am. J. Reprod. Immunol..

[60] Forrester M.A., Wassall H.J., Hall L.S., Cao H., Wilson H.M., Barker R.N., Vickers M.A. (2018). Similarities and differences in surface receptor expression by THP-1 monocytes and differentiated macrophages polarized using seven different conditioning regimens. Cell. Immunol..

[61] Qin Z. (2012). The use of THP-1 cells as a model for mimicking the function and regulation of monocytes and macrophages in the vasculature. Atherosclerosis.

[62] Li L., Wang S., Zou Z., Tao A., Ai Y. (2018). Activation profile of THP-1 derived dendritic cells stimulated by allergen Mal f 1 beyond its IgE-binding ability. Int. Immunopharmacol..

[63] Berges C., Naujokat C., Tinapp S., Wieczorek H., Hoh A., Sadeghi M., Opelz G., Daniel V. (2005). A cell line model for the differentiation of human dendritic cells. Biochem. Biophys. Res. Commun..

[64] Carse S., Lang D., Katz A.A., Schäfer G. (2021). Exogenous Vimentin Supplementation Transiently Affects Early Steps during HPV16 Pseudovirus Infection. Viruses.

[65] Schäfer G., Graham L.M., Lang D.M., Blumenthal M.J., Bergant Marušič M., Katz A.A. (2017). Vimentin Modulates Infectious Internalization of Human Papillomavirus 16 Pseudovirions. J. Virol..

[66] Bolte S., Cordelieres F.P. (2006). A guided tour into subcellular colocalization analysis in light microscopy. J. Microsc..

