# Supplementary material for: Functional Characterisation of Surfactant Protein A as a Novel Prophylactic Means against Oncogenic HPV Infections
Source: Int J Mol Sci. 2024 Jul 14;25(14):7712. doi: 10.3390/ijms25147712 (PMC11277218; doi:10.3390/ijms25147712)
Supplement: Supplementary file 1 [file ijms-25-07712-s001.zip › ijms-3077548-supplementary.pdf]

Supplementary data

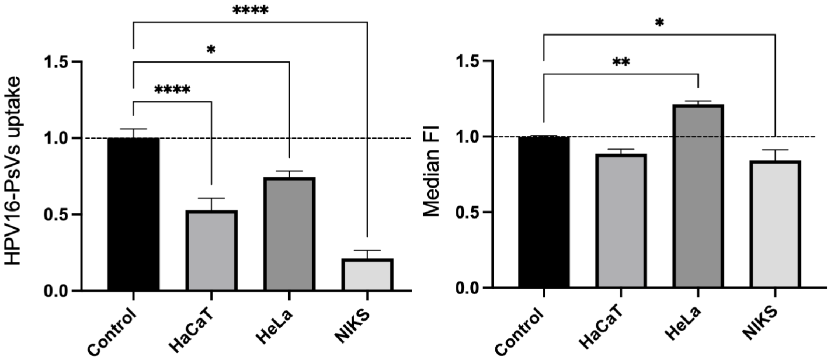

Figure S1. HPV16-PsVs internalisation into HaCaT, HeLa, and NIKS cells is altered by SP-A preincubation. Cells were incubated with HPV16-PsVs as described before acquisition with a BD LSR-Fortessa. A. Fold change of HPV16-PsVs uptake after 1 h into the epithelial cell lines HaCaT, HeLa, and NIKS in the presence of SP-A is depicted with the BSA control set as 1. B. Fold-change of the MFI of internalised HPV16-PsVs in the presence of SP-A is depicted with the BSA control set as 1. Data of three independent experiments are presented relative to uptake of the BSA control group. Statistical significance was determined by two-way ANOVA and Tukey's multiple comparison tests. *= p<0.05; **= p< 0.01; ****= p<0.0001; no symbol denotes not significant.

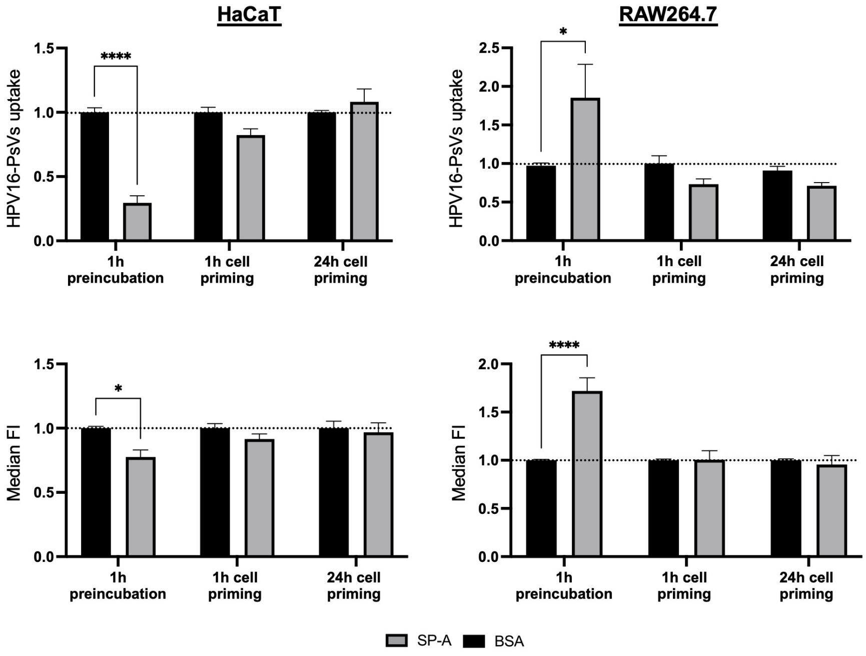

Figure S2. Assessment of HPV16-PsVs preincubation with SP-A versus SP-A priming of cells for modulation of viral internalisation. Three conditions were analysed: 1. Preincubation of HPV16-PsVs with BSA or SP-A in the presence of 5 mM $CaCl_2$ 1 h before infection; 2. Priming of cells with BSA or SP-A in the presence of 5 mM $CaCl_2$ for 1 h prior to HPV16-PsVs infection; 3. Priming of cells with BSA or SP-A in the presence of 5 mM $CaCl_2$ 24 h prior to HPV16-PsVs infection. All cells were harvested 1 h post infection and acquired as described. HPV16-PsVs uptake in the presence of SP-A is depicted in the top graphs and the MFI of internalised HPV16-PsVs is depicted below. Data of three independent experiments are presented relative to uptake of the BSA control group which was set as 1. Statistical significance was determined using two-way ANOVA and Sidak's multiple comparison tests. *=p<0.05; ****= p<0.0001; no symbol denotes not significant.

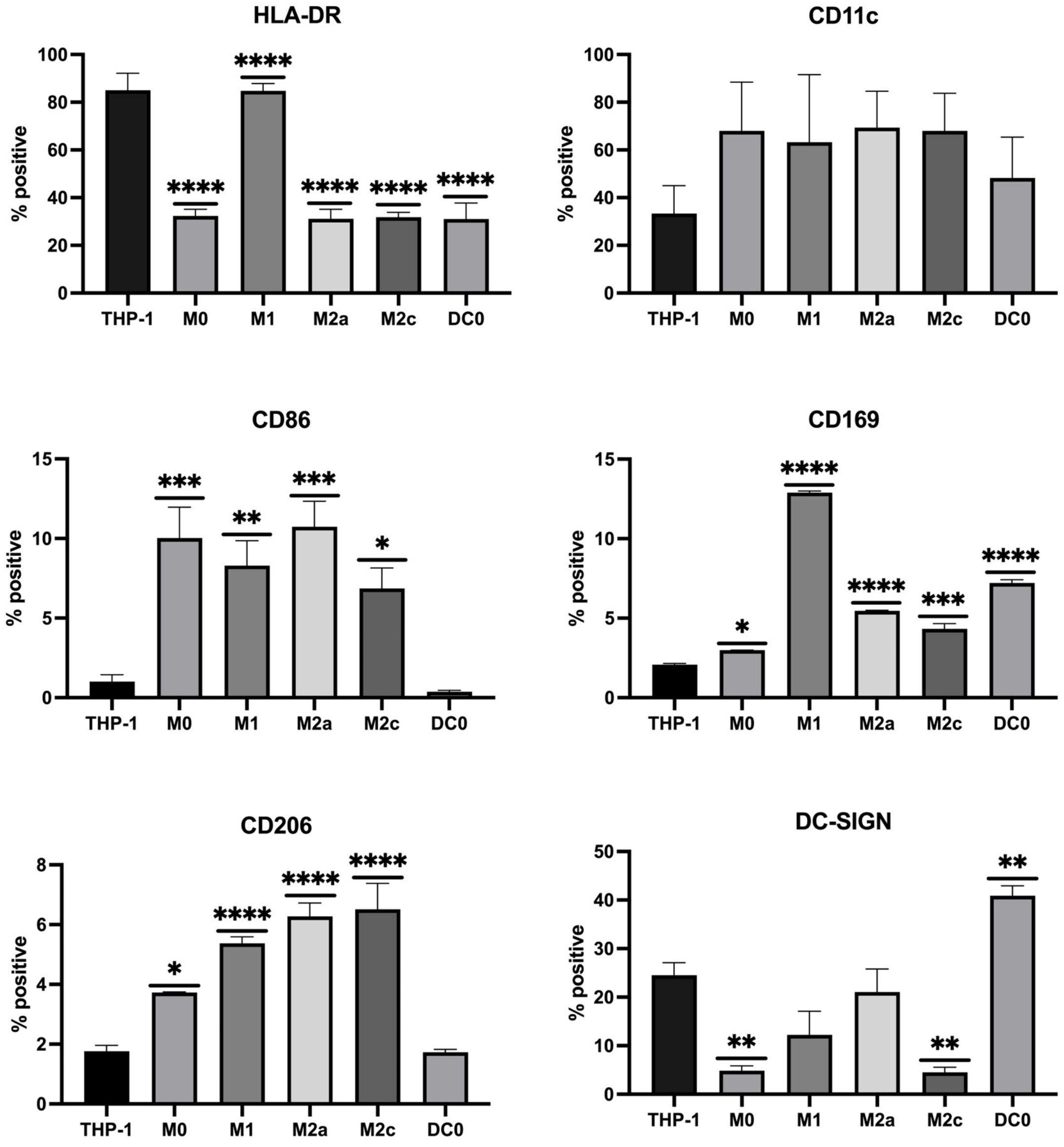

Figure S3. Cell-surface marker expression on THP-1-derived immune cells. Cells were stained as described in Section 4.2. before acquisition with the BD LSRFortessa. Percentage positivity relative to the whole live population for HLA-DR, CD11c, CD86, CD169, CD206 and DC-SIGN are shown. Data are from 3 independent experiments where the mean and SEM are plotted. Statistical significance was determined using one-way ANOVA and Tukey's multiple comparison tests relative to the undifferentiated THP-1 cells. *= $p<0.05$; **= $p< 0.005$; ***= $p<0.001$; ****= $p<0.0001$; no symbol denotes not significant.

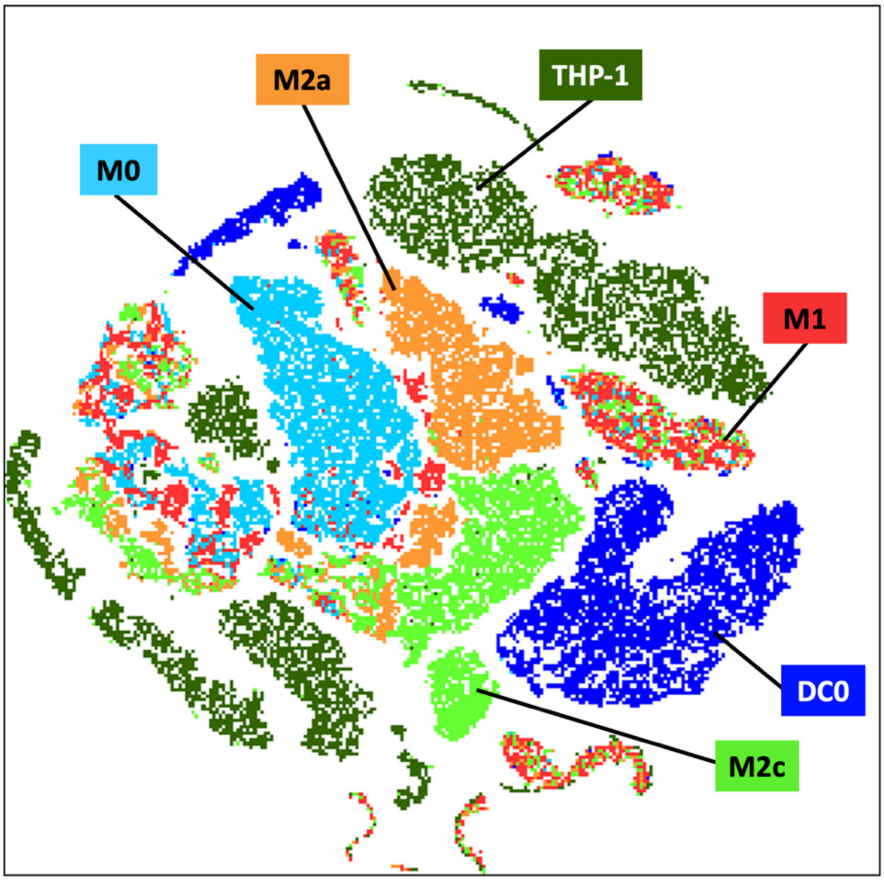

Figure S4. Phenotypic analysis of THP-1-derived immune cells using t-SNE. Dimensionality reduction and clustering analysis of flow cytometry data for the THP-1 cell derived human ICP. Distinct clusters of immune cell populations are color-coded and labelled. The tSNE algorithm was performed in FlowJo (v 10.7.2).

Table S1. Immune modulators that were differentially expressed in the SP-A:HPV16-PsVs group relative to the untreated control in A) THP1 cells and B) DC0 cells. A 0.5-fold change in expression was used as the cut-off value. Shown are mean values obtained from the duplicate dots on the Proteome Profiler™ Human XL Cytokine Array membranes. Proteins are ordered from highest to lowest according to the fold-change in expression of SP-A:HPV16-PsVs relative to the untreated control.

A

Name	Fold expression relative to untreated condition		
	SP-A only	HPV16-PsVs only	SP-A:HPV16-PsVs
VEGF	1.19	2.82	3.28
C5	4.04	1.49	2.97
Apoliprotein A-I	2.43	1.22	2.53
Lipocalin-2	2.04	1.31	1.69
FGF-7	0.97	2.22	1.59
Adiponectin	2.29	0.95	2.09
Growth hormone	0.57	1.09	0.50
GRO-α	0.64	1.02	0.42

B

Name	Fold expression relative to untreated condition		
	SP-A only	HPV16-PsVs only	SP-A:HPV16-PsVs
Cripto-1	5.43	5.22	14.12
EGF	3.45	1.20	5.47
FGF-7	1.58	2.38	5.35
IL-8	1.28	4.03	4.59
FLT3L	2.45	1.67	4.58
CD26	3.03	1.18	4.60
C5	2.48	0.94	4.21
Adiponectin	2.12	0.99	3.45
Apoliprotein A-I	2.89	1.15	3.24
CD30	1.43	1.52	3.20
Angiopoietin-1	1.34	1.68	3.18
G-CSF	1.38	1.36	3.02
IL-1β	1.44	1.07	2.59
LIF	0.91	1.02	2.28
TNFα	1.50	1.67	2.27
CRP	0.91	1.44	2.24
Growth hormone	0.79	1.31	2.23
Angiogenin	1.61	1.15	2.14
CCL20	0.91	1.02	2.12
TIM-3	1.17	1.54	2.09
IL-2	1.32	1.23	2.04
IL-13	0.82	0.93	1.98
CCL3	0.74	1.15	1.94
ICAM-1	1.14	0.75	1.93
IL-15	0.98	1.92	1.92
VCAM-1	1.03	1.39	1.89
PDG	1.20	1.01	1.89
FGF-2	0.88	1.06	1.87

IGFBP-3	1.03	1.01	1.79
IL-3	0.92	1.23	1.79
RBP4	1.37	1.30	1.77
TGFα	1.20	1.42	1.77
IL-12	0.99	0.88	1.74
CD40 ligand	0.99	1.06	1.72
IL-16	0.96	1.69	1.69
IL-22	0.93	1.66	1.66
IL-19	0.82	1.64	1.64
Fas Ligand	0.98	1.16	1.63
IL-24	0.73	1.58	1.58
Thrombospondin-1	1.12	1.18	1.57
TARC	0.92	1.02	1.56
